# COVID-19 in solid organ transplantation patients: A systematic review

**DOI:** 10.6061/clinics/2020/e1983

**Published:** 2020-05-25

**Authors:** Lucas Souto Nacif, Leonardo Y. Zanini, Daniel R. Waisberg, Rafael S. Pinheiro, Flávio Galvão, Wellington Andraus, Luiz Carneiro D'Albuquerque

**Affiliations:** Divisao de Transplante de Figado e Orgaos do Aparelho Digestivo, Departamento de Gastroenterologia, Hospital das Clinicas HCFMUSP, Faculdade de Medicina, Universidade de Sao Paulo, Sao Paulo, SP, BR

**Keywords:** Liver Transplantation, Systematic Review, COVID-19, Humans, Liver Diseases, SARS-CoV-2, 2019-nCoV, Solid Organ Transplant Recipient

## Abstract

Coronavirus disease (COVID-19) rapidly progresses to severe acute respiratory syndrome. This review aimed at collating available data on COVID-19 infection in solid organ transplantation (SOT) patients.

We performed a systematic review of SOT patients infected with severe acute respiratory syndrome coronavirus 2 (SARS-CoV-2). The MEDLINE and PubMed databases were electronically searched and updated until April 20, 2020. The MeSH terms used were “COVID-19” AND “Transplant.”

Thirty-nine COVID-19 cases were reported among SOT patients. The median interval for developing SARS-CoV-2 infection was 4 years since transplantation, and the fatality rate was 25.64% (10/39). Sixteen cases were described in liver transplant (LT) patients, and the median interval since transplantation was 5 years. The fatality rate among LT patients was 37.5% (6/16), with death occurring more than 3 years after LT. The youngest patient who died was 59 years old; there were no deaths among children. Twenty-three cases were described in kidney transplant (KT) patients. The median interval since transplantation was 4 years, and the fatality rate was 17.4% (4/23). The youngest patient who died was 71 years old. Among all transplant patients, COVID-19 had the highest fatality rate in patients older than 60 years : LT, 62.5% *vs* 12.5% (*p*=0.006); KT 44.44% *vs* 0 (*p*=0.039); and SOT, 52.94% *vs* 4.54% (*p*=0.001).

This study presents a novel description of COVID-19 in abdominal SOT recipients. Furthermore, we alert medical professionals to the higher fatality risk in patients older than 60 years. (PROSPERO, registration number=CRD42020181299)

## INTRODUCTION

Coronavirus disease 2019 (COVID-19) is caused by the novel coronavirus, severe acute respiratory syndrome coronavirus-2 (SARS-CoV-2) (1,2). The first case of this disease was reported in Wuhan, Hubei province, China, in December 2019 (3). Since March 2020, COVID-19 has spread worldwide and has become a public health emergency and a pandemic of international concern (4).

COVID-19 is extremely contagious and, in certain cases, may rapidly progress to severe acute respiratory syndrome. An unexpectedly high rate of SARS-CoV-2 infection has been observed worldwide. An age gradient in the risk of death was identified, and patients older than 60 years were reported to have a higher mortality rate (5,6), with a case fatality rate close to 8-10% (5,6). Moreover, the presence of comorbidities results in increased mortality, with multiorgan dysfunction (1,2).

Data on COVID-19 in solid organ transplant (SOT), liver transplant (LT), and kidney transplant (KT) patients are scarce (7). Mazzafero et al. (8) reported a novel experience with several COVID-19 cases at an Italian transplant center in Lombardy (8). Another case series from Italy showed that children who had received LTs were not at a higher risk for severe SARS-CoV-2 infection despite being immunosuppressed (9).

Transplant societies and guidelines were initially established to decrease the risk of infection and protect health care professionals. Recipients and donors may be at risk of being carriers of SARS-CoV-2 or contracting active COVID-19 through contact with patients who have COVID-19 or through exposure to SARS-CoV-2 (10).

Insufficient data on infected SOT recipients are available, particularly data regarding the management of immunosuppressants and fatality rates. This review aimed to collate the available data that address the management of COVID-19 infection in abdominal SOT patients.

## METHODS

### Study identification

A systematic review of the literature on COVID-19 and transplantation was carried out. The MEDLINE and PubMed databases (http://www.ncbi.nlm.nih.gov/pubmed) were electronically searched and updated until April 20, 2020. The MeSH terms used were “COVID-19” (entire related MeSH terms: 2019 novel coronavirus, SARS-CoV-2 infection, 2019-nCoV infection) AND “transplant.” The electronic bibliographic database included MEDLINE-PubMed, EMBASE, Cochrane Library, and Web of Science.

The terms and MeSH terms for the PubMed database search were developed with the PICO structure: patient, intervention, comparison or control, and outcome (PICO). The terms for each group were combined with the “OR” operator. The results of the search terms forming the “P” (Patients) group were combined with those of search terms forming the “I” (Intervention) group, with “AND,” and for exclusion terms, with “NOT.”

Participants/population: Adults and children who were abdominal organ recipients and were diagnosed as having COVID-19. Intervention(s), exposure(s): Adults and children who were abdominal SOT recipients and tested positive for COVID-19 that progressed to severe acute respiratory syndrome. Comparator(s)/control: The control group was selected from the group of patients who were not exposed to SARS-CoV-2. We evaluated other groups of LT and KT patients and various epidemiologic groups such as those matched for age, sex, and comorbidities. Context Main outcome(s): Survival after SARS-CoV-2 infection and outcomes related to medical management, as a potential therapy to this infection in this population; establishing guidelines and programs to better treat this population.

This systematic review was registered in the international database of prospectively registered systematic reviews (PROSPERO, registration number=CRD42020181299). The review protocol can be accessed online via the PROSPERO website (https://www.crd.york.ac.uk/prospero/). The Preferred Reporting Items for Systematic Reviews and Meta-Analysis (PRISMA) checklist was adhered to when preparing this manuscript (11,12,13). The review methodology followed the recommendations published by PRISMA (11,12,13).

### Study selection

#### Inclusion and exclusion criteria

Selection criteria were used within the research question of the PICO structure. All studies evaluated were written in English.

Case reports, letters to the editor, clinical randomized controlled trials, non-randomized controlled trials, reviews, consensus articles, and protocol studies were included. Studies on organs other than the liver and kidneys, those on tissues transplants, those on a novel therapy for COVID-19, those on the impact of COVID-19 on the transplant system, those on vaccine research, those involving patients on hemodialysis, those on the clinical manifestation of COVID-19, epidemiologic studies, those on elective or non-transplant surgical procedures, and those on immunosuppression protocols that were unrelated to abdominal SOT and COVID-19 were excluded.

#### Study data extraction

Data extraction was carried out independently by two researchers, using the text, tables, and figures of the original published articles. The quality of the studies selected and the selection methods were evaluated by two independent researchers (LSN and LYZ). In the case of a disagreement, the researchers held a consensus meeting to reach a final decision.

#### Statistical analysis

Quantitative and qualitative variables were presented as number and percentage, median and range, or mean and standard deviation. The COVID-19 prevalence and fatality rates of patients who were older than 60 years were calculated and compared. A Mann-Whitney *U* test was used to compare independent samples, and *p*<0.05 was considered significant. All tests were performed using IBM SPSS 25 software, with α=0.05 and a 95% confidence interval.

The data were generated using Review Manager Version 5.3 software provided by the Cochrane Collaboration (RevMan; The Cochrane Collaboration, The Nordic Cochrane Centre, Copenhagen, Denmark).

## RESULTS

### Study selection

**Figure 1:**
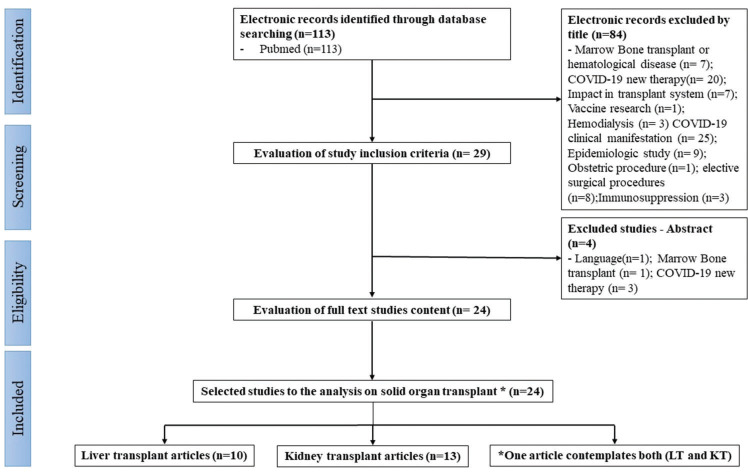
Flow diagram of the systematic literature search according to the PRISMA statement. *Kidney transplant (KT) and liver transplant (LT).

The literature search revealed 113 articles, of which 24 articles were selected and analyzed in this review. Figure 1 depicts the flow diagram of the systematic literature search, according to the PRISMA statement, and the selection criteria for the articles.

We did not find well-designed randomized control trials, cohorts, or prospective or retrospective studies. Most articles were case series, letters to the editor, and editorials.

Data extraction and synthesis were performed specifically using articles on SOT with COVID-19 infection, that is, 24 articles. There were 11 articles regarding Liver transplantation (Table 1), 14 articles on Kidney transplantation (Table 2), and one article on SOT as presented with liver and KT.

### Solid organ transplant patients

Twenty-four articles on SOT patients were selected. Thirty-nine cases were related to SOT and COVID-19 infection. The median interval since transplantation to COVID-19 infection was 4 years (range, 0.01 - 30.10). The overall fatality rate in solid organ transplant patients was 25.64% (10/39), and the fatality rate was 52.94% in patients older than 60 years (*p*=0.001; Table 3).

### Liver transplant patients

From the 11 articles included in the analysis of LT patients infected by SARS-CoV-2, seven were case reports/correspondences (8,9,), 3 were consensuses (19-21), and one article was a case series on overall SOT (7). The LT group included a total of 16 cases, with a median interval since transplantation of 5.95 years (range, 0.01-26.5). These cases involved transplants in pediatric and adult patients, with no reported deaths in children. Tacrolimus administration was discontinued in two cases, with satisfactory evolution of these patients; however, therapy with methylprednisolone was maintained (Table 1). The LT case fatality rate was 37.5% (6/16). Six patients evolved to death, more than 3 years after LT, with the youngest being 59 years of age (Table 1 and Table 3).

### Kidney transplant patients

Fourteen articles were included in the analysis of KT patients with COVID-19. Among these, six were case reports/case series (22-27), five were correspondences (10,), one was a guideline (32), one an editorial (33), and one was a case series on SOT (7). Twenty-three KT cases were described. The median interval since transplantation was 4 years (range, 0.25-30.1). The fatality rate among KT patients was 17.4% (4/23), and the youngest patient who died was 71 years. Immunosuppressive therapy was reduced or suspended in 13 cases, with good clinical evolution in eight patients (Table 2 and 3).

## DISCUSSION

The transplant society’s recommendations and guidelines during the COVID-19 pandemic, with insufficient evidence, consider LT and KT as safe procedures. The transplant team should discuss the real risks and benefits of the procedure and of immunosuppression therapy. On the basis of the scarce data on SARS-CoV-2 infection, it is suggested that therapy for COVID-19 patients be individualized. This review provided data on SOT and COVID-19 infection.

In their study, Mazzafero et al. (8) reported that three patients with severe COVID-19 among 111 long-term LT survivors (who had undergone transplantation more than 10 years ago) died. The post-transplant course had been uneventful for all three patients, and their immunosuppressive regimen had been tapered gradually, with very low trough concentrations of calcineurin inhibitors (two patients receiving ciclosporin and one receiving tacrolimus) (8).

D’Antiga et al. (9) reported a case series from a single center in Italy, wherein children who received LTs were not at increased risk for severe SARS-CoV-2 infection compared with the general population despite being immunosuppressed (9). Nevertheless, this series was biased as it involved the study of a population of children, which has a lower incidence rate of SARS-CoV-2 infection.

The American Association for the Study of Liver Diseases suggests that immunosuppression should not be reduced or stopped in asymptomatic LT recipients (21). Immunosuppression, despite its immunomodulatory effect, did not seem to increase the risk of severe COVID-19 disease in transplantation patients. Given that a reactive innate immune response might be responsible for the severe clinical manifestations of SARS-CoV-2 infection, immunosuppression might be protective against COVID-19; however, this needs further clarification.

Unrecognized COVID-19 infection among transplantation recipients largely increases the potential of development of severe immune suppression and postsurgical infection, which may lead to multisystem organ damage or death. A donor with unidentified COVID-19 infection may also spread the virus to multiple recipients. The therapeutic paradox is compelling in such patients: insufficient immunosuppression results in graft loss due to rejection, whereas excessive immunosuppression results in severe infection. Strict screening protocols for organ transplant recipients and donors, aimed at identifying carriers of SARS-CoV-2, must be developed to aid in reducing further transmission (14). However, the scarce data must be better analyzed with more prolonged follow-up, and evaluations should be carried out in recipients with COVID-19 infection.

Fernandez-Ruiz et al. (7) reported on 18 SOT recipients with COVID-19. The majority of patients were KT recipients (44.4% [8/18]), followed by LT (33.3% [6/18]) and heart transplant recipients (22.2% [4/18]). The median patient age was 71.0±12.8 years, with a predominance of male patients (77.8% [14/18]), and the median interval since transplantation was 9.3 years. The case fatality rate was 27.8% (5/18). Their study suggests that SARS-CoV-2 infection had a severe course in SOT recipients (7). Moreover, this finding differs from those of other publications, which report similar results in non-transplanted patients with COVID-19 infection. However, the current study, which analyzed overall SOT cases, found that the median interval from transplantation to COVID-19 infection was 4 years, and the case fatality rate was 25.64% (10/39). Therefore, we demonstrated that SOT populations have a higher mortality risk than that in non-transplanted populations.

Moreover, Verity et al. (5) demonstrated an age gradient in the risk of death in a COVID-19-infected population, which showed that early estimates indicate the fatality ratio (5). According to their findings, there was an increase in the severity of COVID-19 with age in patients older than 60 years, along with a higher fatality rate (6). Moreover, in this study, we observed a significant increase in fatality risk among SOT patients older than 60 years.

The main limitations of this study were that only case series and editorials on the topic were found in the literature. To the best of our knowledge, the number of cases with patients who had undergone SOT and were infected with COVID-19 is considerably low. Further, only this review was registered in the PROSPERO database (https://www.crd.york.ac.uk/prospero/). However, there are several ongoing clinical trials on COVID-19 (https://clinicaltrials.gov/ct2/results?cond=COVID-19). Further data is warranted for more definitive recommendations. The main contributions of this systematic review are the novel description of COVID-19 infection in SOT recipients (LT and KT), especially in terms of the management of the disease and immunosuppression therapy, and the fact that it alerts medical professionals to the higher fatality risk in SOT patients older than 60 years.

## CONCLUSION

This study presents a novel description of COVID-19 infection in abdominal SOT recipients. Furthermore, we alert medical professionals to the higher fatality risk in the population of patients older than 60 years.

## AUTHOR CONTRIBUTIONS

All authors have approved the final draft of the submitted manuscript. Nacif LS was responsible for the study conception and design, data collection, analysis and interpretation, manuscript writing, and literature search. Zanini LY, Waisberg DR and Pinheiro RS were responsible for the study conception and design, data collection, analysis and interpretation, and critical revisions. Galvão F, Andraus W and D'Albuquerque LC were responsible for the study conception, interpretation and critical revisions.

## Figures and Tables

**Table 1:** COVID-19 infection in liver transplant patients.

Article	Patients	Clinical manifestation	Laboratorial exams	Treatment	Outcome
Zhong et al. (16) and Qin et al. (14) (Case report)	37 years old Male VHB+HCC 9 days after LT FK+MPA	-Fever (38.6°C) -SpO_2_ 94% -Weakness -Abdominal discomfort -Sleep disorders	-WBC 2.46×10^9^/L -Platelets 74×10^9^/L -Lymphs 0.48×10^9^/L -ALT 240 U/L -TBIL 38.9 μmol/L -RT-PCR positive for SARS-CoV-2 -CT scan showed bilateral glass density lesions	-UCI -Tacrolimus use was suspended -Administrated intravenous methylprednisolone (40 mg; 12/12h) -Oseltamivir -Cefoperazone -Sulbactam sodium	-After 2 days in UCI, satisfactory evolution -However, after 14 days of FK suspension, presented with possible graft dysfunction -Patient underwent MPA pulse therapy with satisfactory evolution
Huang et al. (17) (Case report)	59 years old Male VHB+HCC 3 years after LT FK+MMF	Three days of fever (40°C), cough, chills, fatigue, and diarrhea. HR of 112 beats/min, jaundice, splenomegaly, and ascites	-WBC 3.2×10^9^/L, -Lymphs 0.7×109/L -CRP 35.1 mg/L, -TBIL 83.9 μmol/L, -ALT 60 U/L -GGT 1087 U/L -RT-PCR positive for SARS-CoV-2 -CT scan showed bilateral glass density lesions	-UCI -Nebulized α- interferon -Umifenovir -Lopinavir/ritonavir -Piperacillin and tazobactam (empirical) -Required invasive ventilation (day 4)	-On the 12^th^ day of hospitalization, patient had positive cultures of *Candida albicans* and *Pseudomonas aeruginosa* -Worsening of hepatic and renal function -Plasma, immunoglobulin, blood transfusion, and hemodialysis were used -On the 33rd hospitalization day, RT-PCR was negative for SARS-CoV-2, but the patient evolved to death on the 47th day
Bin et al. (18) (Case report)	50 years old Male VHB 3 years after LT FK	-Fever (39.6°C) but initially low fever -Progressive dyspnea	-WBC 5.9×10^9^/L -CRP 32.1 mg/L -RT-PCR positive for SARS-CoV-2 -CT scan showed bilateral glass density lesions	-Discontinued FK and received systemic MPA -Cefoperazone (prophylactic) -Umifenovir -Lopinavir/ritonavir -Immunoglobulin -Nebulized α- interferon	-Patient had a satisfactory clinical evolution, with no complications and no liver dysfunction
Bhoori et al. (8) (Correspondence)	3 patients, >65 years old All male >10 years of LT Two patients receiving ciclosporin and one FK	Hospital admission with community-acquired pneumonia and quickly developed severe respiratory distress syndrome	Positive nasopharyngeal swab test for SARS-CoV-2	-UCI -Required invasive ventilation	All three patients evolved to death 3-12 days after the first pneumonia symptoms manifested
D’Antiga L (9) (Correspondence)	3 liver transplant children (From 700 LT)	None of the three patients had a clinical manifestation	Positive nasopharyngeal swab test for SARS-CoV-2	None	The three transplanted children did not develop any symptoms of COVID-19
Lagana S. et al. (15) (Case report)	6 female months children Biliary atresia LDLT COVID-19 infection manifestation 2 days after LT	-Fever -Dyspnea (employed CPAP) -Diarrhea	-RT-PCR positive for SARS-CoV-2 -Liver biopsy: mixed inflammatory infiltrate (lymphs) -Interlobular bile ducts with lymphocytic cholangitis	-Hydroxychloroquine	Patient still hospitalized, but with mild respiratory symptoms
Fernández-Ruiz et al. (7) (Case series)	Case 1 -63 years old -Male -COVID-19 infection manifestation 7.9 years after LT HBV+HCV+HCC -Everolimus	-Fever -SpO_2_ 96% -Dyspnea -Cough -Diarrhea -Myalgia -Diarrhea	-Unifocal consolidation on chest X-Ray -Positive nasopharyngeal swab test for SARS-CoV-2	-Hydroxychloroquine -Lopinavir/ritonavir -Suspension of everolimus and substitution to FK +MMF	-Patient had a satisfactory clinical evolution, with no complications
	Case 2 -72 years old -Male -COVID-19 infection manifestation 5.5 years after LT Cryptogenic cirrhosis Everolimus+MMF	-Fever -SpO_2_ 95% -Dyspnea -Cough	-Multifocal consolidation on chest X-Ray -Positive nasopharyngeal swab test for SARS-CoV-2	-Hydroxychloroquine -Lopinavir/ritonavir -Interferon-β -Substitution of Everolimus+MMF to FK	-Patient had worsening respiratory function and renal failure. Evolved to death after 7 days
	Case 3 -79 years old -Female -COVID-19 infection manifestation 15.3 years after LT -HCV+HCC -Prednisone+AZA+ Everolimus	-SpO_2_ 96% -Dyspnea -Cough -Malaise -Diarrhea	-Bilateral consolidation on chest X-Ray -Positive nasopharyngeal swab test for SARS-CoV-2	-Hydroxychloroquine -Interferon-β -Immunosuppressive drugs maintained	-Patient had a satisfactory clinical evolution, with no complications
	Case 4 -73 years old -Male -COVID-19 infection manifestation 16.4 years after LT -HBV -MMF	-Fever -SpO_2_ 76% -Dyspnea -Cough -Malaise	-Multifocal consolidation on chest X-Ray -Positive nasopharyngeal swab test for SARS-CoV-2	-ICU -Mechanical ventilation -Suspended MMF	-Patient had severe respiratory dysfunction and evolved to death in 24 days
	Case 5 -76 years old -Female -COVID-19 infection manifestation 26.5 years after LT -HCV -FK	-Fever -SpO_2_ 98% -Thoracic pain	-Positive nasopharyngeal swab test for SARS-CoV-2	-Hydroxychloroquine -FK maintained	-Patient had a satisfactory clinical evolution, with no complications
	Case 6 -46 years old -Female -COVID-19 infection manifestation 6.4 years after LT -Acute liver failure -FK	-SpO_2_ 100% -Diarrhea	-Positive nasopharyngeal swab test for SARS-CoV-2	-FK maintained	-Patient had a satisfactory clinical evolution, with no complications

ALT, alanine aminotransferase; CT, computed tomography; CRP, C-reactive protein; FK, tacrolimus; GGT, gamma-glutamyl transpeptidase; HCC, hepatocellular carcinoma; HR, heart rate; LT, liver transplantation; Lymphs, lymphocyte; MMF, mycophenolate mofetil; MPA, methylprednisolone; RT-PCR, real-time polymerase chain reaction; SARS-CoV-2, Severe Acute Respiratory Syndrome Coronavirus-2; SpO_2_, peripheral capillary oxygen saturation; TBIL, total bilirubin; UCI, intensive care unit; VHB, hepatitis B virus; WBC, white blood cells.

**Table 2:** COVID-19 infection in kidney transplant patients.

Article	Patients	Clinical manifestation	Laboratory examination	Treatment	Outcome
Bussalino et al. (22) (Case report)	-32 years old -Male -3 years KT -Deceased donor -ESRD etiology was unknown -FK+MMF+Prednisone	-Three days of fever (38°C) -SpO2 97% -Dyspnea -Non-productive cough	-CRP 47.9 mg/L -PCT 0.33 μg/L -Creatinine 2.6 mg/dl -GFR 31 ml/min/1.73m^2^ -RT-PCR positive for SARS-CoV-2	-Prednisone dose was increased to 15 mg/day -FK+MMF maintained -hydroxychloroquine -Oseltamivir -Ceftaroline -Oxygen administration by nasal catheters	-Patient had a satisfactory clinical evolution -Renal function remained similar to that at admission
Marx et al. (28) (Correspondence)	-58 years old -Male -3 years KT -Renal vein thrombosis -Balatacept+MMF+Prednisone	-Fever (38°C) -Dyspnea -Cough -History of contact with a person who received a COVID-19 diagnosis	-RT-PCR positive for SARS-CoV-2 -Pulmonary ground-glass opacities on CT	-Belatacept and MMF suspended at hospital admission	-Patient had a satisfactory evolution, without the necessity of mechanical ventilation or supplementary oxygen -Introduced cyclosporine on the 7^th^ hospitalization day
Bartiromo et al. (23) (Case report)	-36 years old -Female -First KT in 1993 -Second KT in 1995 (Cadaveric donor) -Senior-Loken syndrome -FK+Corticoesteroids	-Afebrile (36.3°C) -SpO2 97% -Fatigue -Dry cough -Coryza -History of contact with her mother who received a COVID-19 diagnosis	-CRP 67 mg/L -Creatinine 2.29 mg/dl -GFR 27 ml/min -RT-PCR positive for SARS-CoV-2	-Lopinavir/ritonavir* -Hydroxychloroquine -Ceftriaxone *On the 2^nd^ hospitalization day, patient presented with nausea and diarrhea, so the therapy with lopinavir/ritonavir was replaced with darunavir/cobicistat	- On the 4th hospitalization day, the patient presented with abdominal pain, nausea, and diarrhea. -Had a good clinical evolution -Medical release on the 9th hospitalization day
Ning et al. (24) (Case report)	-29 years old -Male -2 years KT (Living donor) -Cyclosporine+MMF+methylprednisolone	-Fever (37.7°C) -Without other symptoms in admission -During hospitalization (2^nd^ day), patient presented with nasal stuffiness, hyporexia, nausea, and vomiting	-First test RT-PCR negative for SARS-CoV-2 -Pulmonary ground-glass opacities on CT -Second test RT-PCR positive for SARS-CoV-2 (3rd hospitalization day)	-TMP-SMX (admission) -Immunosuppressive treatment was maintained -Lopinavir/ritonavir (after RT-PCR test was positive) -Probiotics	-On the 3^rd^ hospitalization day, patient presented with dizziness, nausea, vomit, oliguria, and hematuria -Laboratory tests revealed elevated creatinine levels and hyponatremia -Patient received fluids and had better renal function -Medical release on the 12th hospitalization day
Wang et al. (10) (Correspondence)	-49 years old -Male -2 years KT -Cyclosporine+MMF+ Prednisone	-Fever for six days -Without other symptoms in admission	-WBC 7.18×10^9^/L, -Lymphs 0.59×10^9^/L -CRP 22.73 mg/L -PCT 0.36 μg/L -Creatinine 1.44 mg/dl -RT-PCR positive for SARS-CoV-2 -Pulmonary ground-glass opacities on CT	-Nebulized α- interferon -Immunosuppressive treatment was maintained -Ribavirin -Lopinavir/ritonavir -Methylprednisolone -Oxygen administration by nasal catheters	-Patient had a good clinical evolution, without an impact on renal function
	Case 1 -38 years old -Male -3 months KT (Deceased donor)	-Fever (38.9°C) -SpO2 99% -Cough	-WBC 4.73×10^9^/L, -Lymphs 0.59×10^9^/L -CRP 6.68 mg/L -Creatinine 1.10 mg/dl -RT-PCR positive for SARS-CoV-2	-Antiviral therapy (oseltamivir or arbidol)	-Patient had a good clinical evolution, without the necessity of mechanical ventilation
	Case 2 -64 years old -Male -4 years KT (Deceased donor)	-Fever (38.3°C) -SpO2 96% -Cough -Sputum -Fatigue	-WBC 17.67×10^9^/L, -Lymphs 0.55×10^9^/L -CRP 337.11 mg/L -Creatinine 4.65 mg/dl -RT-PCR positive for SARS-CoV-2	-Antiviral therapy (oseltamivir or arbidol) -Cefixime	-Patient had anuria, persistent fever, normal CT chest, and an elevation of creatinine levels - Realized methylprednisolone pulse therapy, due rejection suspect -Had a good clinical evolution
Zhang et al. (25) (Case Series)	Case 3 -37 years old -Female -5 months KT (Deceased donor)	-Fever (39°C) -SpO2 99% -Cough	-WBC 5.67×10^9^/L, -Lymphs 0.31×10^9^/L -CRP 9.77 mg/L -Creatinine 1.54 mg/dl -RT-PCR positive for SARS-CoV-2	-Antiviral therapy (oseltamivir or arbidol) -IV immunoglobulin	-Patient had a good clinical evolution, without the necessity of mechanical ventilation
	Case 4 -47 years old -Male -11 months KT (Deceased donor)	-Fever (39.8°C) -SpO2 98% -Cough -Sputum -Fatigue	-WBC 3.99×10^9^/L, -Lymphs 0.51×10^9^/L -CRP 13.38 mg/L -Creatinine 1.66 mg/dl -RT-PCR positive for SARS-CoV-2	-Antiviral therapy (oseltamivir or arbidol)	-Patient had a good clinical evolution, without the necessity of mechanical ventilation
	Case 5 -38 years old -Male -3 years KT (Deceased donor)	-Fever (39.1°C) -SpO2 97% -Cough -Sputum -Fatigue	-WBC 6.44×10^9^/L, -Lymphs 0.91×10^9^/L -CRP 33.72 mg/L -Creatinine 1.53 mg/dl -RT-PCR positive for SARS-CoV-2	-Antiviral therapy (oseltamivir or arbidol)	-Patient had a good clinical evolution, without the necessity of mechanical ventilation
Chen et al. (26) (Case report)	-49 years old -Male -6 years KT (Deceased donor) -FK+MMF -Prednisolone	-Fever (38.6°C) -Hyporexia -Dry cough	-WBC 3.44×10^9^/L, -Lymphs 0.43×10^9^/L -CRP 74.34 mg/L -Creatinine 1.84 mg/dl -RT-PCR positive for SARS-CoV-2 -Pulmonary ground-glass opacities on CT	-MMF and prednisolone were suspended -Methylprednisolone -Ribavirin -Immunoglobin -Moxifloxacin	-Patient had worsening blood oxygen saturation, and oxygen inhalation was initiated. -Had a satisfactory clinical evaluation
Seminari et al. (27) (Case report)	-50 years old -Male -First KT 1993, failure 2008 -Second KT 2016 (4 years) -IgA nephropathy -FK+MMF	-Fever (37.3°C) -Cough	-WBC 3.2×10^9^/μL -Lymphs 0.6×10^9^/μL -RT-PCR positive SARS-CoV-2 -Chest x-ray showed interstitial lesions	-Immunosuppressive treatment was maintained -Lopinavir/ritonavir -Ceftriaxone	-Patient had a good clinical evolution, without the necessity of mechanical ventilation
Gandolfini et al. (29) (Correspondence)	Case 1 -75 years old -Male -10 years KT (Deceased donor) -FK+MMF -Prednisolone	-Fever (38-39°C) -Cough -Myalgia -Dyspnea	-WBC 6.56×10^9^/L, -Lymphs 0.80×10^9^/L -CRP 180 mg/L -PCT 1.29 ng/ml -Creatinine 2.2 mg/dl -RT-PCR positive for SARS-CoV-2 -Pulmonary ground-glass opacities on CT	-FK+MMF was discontinued -Lopinavir/ritonavir -Hydroxychloroquine	-Patients presented with significant worsening of respiratory function and evolved to death
	Case 2 -52 years old -Female -8 months KT (Deceased donor) -FK+MMF -Prednisolone	-Fever (38-39°C) -Cough -Fatigue -Dyspnea -Diarrhea	-WBC 2.54×10^9^/L, -Lymphs 0.11×10^9^/L -CRP 158 mg/L -PCT 0.98 ng/ml -Creatinine 2.4 mg/dL -RT-PCR positive for SARS-CoV-2 -Pulmonary ground-glass opacities on CT	-FK+MMF was discontinued -Dorunavir/cobicistat -Hydroxychloroquine	-Patient received non-invasive oxygen therapy -Had a good clinical evaluation
Guillen et al. (30) (Case report)	-50 years old -Male -3^rd^ KT (4 years ago) -IgA nephropathy -FK+everolimus+ prednisone	*1^st^ day -Fever (38.2°C) -Vomiting -Signs of dehydration *5^th^ day -Fever (37.4°C) -Crackles in the right lower lung -Conjunctivitis of the left eye	*1^st^ day -WBC 8.58×10^9^/L -CRP<0.50 mg/dL -Creatinine 1.6 mg/dL, -GFR 50 mL/min *5^th^ day -WBC 10.15×10^9^/L -CRP 13.2 mg/dL -Creatinine 1.6 mg/dL, -GFR 50 mL/min -RT-PCR positive for SARS-CoV-2	-FK+Everolimus was discontinued -Lopinavir/ritonavir -Hydroxychloroquine -Ceftaroline+meropenem	-Patient had worsening respiratory function, and mechanical ventilation was initiated. -Until the publication of this article, the patient remains in intensive respiratory support
Fernández-Ruiz et al. (7) (Case series)	Case 1 -78 years old -Male -8.3 years of KT -Polycystic kidney disease -Prednisone+FK	-Fever -SpO_2_ 89% -Dyspnea	-Unilateral consolidation on chest X-ray -RT-PCR positive for SARS-CoV-2	-Lopiravir/ritonavir -Reduction of FK doses	-Patient had respiratory failure and atrial fibrillation -Evolved to death after 5 days
	Case 2 -73 years old -Male -1.8 years of KT -Hypertensive nephropathy -Prednisone+FK+MPA	-Fever -SpO_2_ 94% -Dyspnea -Cough	-Unilateral consolidation on chest X-ray -RT-PCR positive for SARS-CoV-2	-Lopinavir/ritonavir -Reduction of FK doses -Suspended prednisone+MPA	-Patient had myopericarditis -Had a satisfactory clinical evolution
	Case 3 -80 years old -Male -3.8 years of KT -Hypertensive nephropathy -Prednisone+FK+MPA	-SpO_2_ 90% -Dyspnea -Cough -Hyporexia -Myalgia	-RT-PCR positive for SARS-CoV-2	-Lopinavir/ritonavir -Hydroxychloroquine -Reduction of FK doses -Suspended MPA	-Patient had worsening respiratory function -Evolved to death after 16 days
	Case 4 -71 years old -Female -6 years of KT -Unknown cause of renal failure -Prednisone+FK+MPA	-Fever -SpO_2_ 97% -Dyspnea -Cough -Sore throat	-Bilateral interstitial pneumonia -RT-PCR positive for SARS-CoV-2	-Lopinavir/ritonavir -Hydroxychloroquine -Suspended MPA+prednisone	-Patient evolved to death after 16 days
	Case 5 -71 years old -Male -30.1 years of KT -Polycystic kidney disease -FK	-Fever -SpO_2_ 100% -Epigastric pain	-RT-PCR positive for SARS-CoV-2	-Hydroxychloroquine -Intravenous immunoglobulin -Reduction of FK doses	-Patient showed an improvement in abdominal pain and was discharged home but returned to the hospital due to clinical worsening
	Case 6 -76 years old -Male -14.8 years of KT -IgA nephropathy -Prednisone+MMF+rapamycin	-Fever -SpO_2_ 96% -Rhinorrhea	-Bilateral interstitial pneumonia -RT-PCR positive for SARS- CoV-2	-Hydroxychloroquine -Suspended MMF	-Patient had a good clinical evolution, without the necessity of mechanical ventilation
	Case 7 -39 years old -Male -16.8 years of KT -Polycystic kidney disease -Prednisone+FK+ Everolimus	-Fever -SpO_2_ 100% -Myalgia	-RT-PCR positive for SARS- CoV-2	-Hydroxychloroquine -Suspended FK+everolimus	Patient had worsening respiratory function but showed mild radiological improvement
	Case 8 -65 years old -Male -6.5 years of KT -Chronic interstitial nephritis - Prednisone+FK+MPA	-Fever -SpO_2_ 93% -Myalgia -Dyspnea -Cough	-Unilateral focal consolidation on chest X-ray -RT-PCR positive for SARS- CoV-2	-Lopinavir/ritonavir -Hydroxychloroquine -Reduced doses of MPA and FK	-Patient had a positive clinical evolution

**Note:** CRP, C-reactive protein; CT, computed tomography; ESRD, end-stage renal disease; FK, tacrolimus; AZA, azathioprine; KT; kidney transplantation; Lymphs, lymphocyte; MMF, mycophenolate mofetil; PCT, procalcitonin; RT-PCR, real-time polymerase chain reaction; SpO_2_, peripheral capillary oxygen saturation; UCI, intensive care unit; WBC, white blood cells.

**Table 3:** COVID-19 infection in SOT, LT, and KT patients. Overall parameters and prevalence/fatality rate in COVID-19 in SOT, liver and kidney patients.

	OVERALL PARAMETERS			
Parameters	Liver transplant	Kidney transplant	Overall SOT	*p*-value
Cases (N)	16	23	39	-
Age (years)	55.25 (±23.8)	54.65 (±16.42)	54.65 (±16.42)	0.603
	61 (0.5-79)	50 (29-80)	52 (0.5-80)	
Time after transplant to COVID-19 (years)	8.40 (±8.50)	6.79 (±7.84)	7.28 (±7.95)	0.686
	5.95 (0.01-26.5)	4 (0.25-30.1)	4 (0.01-30.10)	
Death (N)	6	4	10	0.157
Fatality rate (%) (death/total)	37.5%	17.39%	25.64%	0.157
Fatality rate ≥60 years old (%)	62.5%	44.44%	52.94%	0.457

	PREVALENCE AND FATALITY RATE (%) (≥60 years old vs <60 years old)		
	Age ≥60 years old	Age <60 years old	*p*-value
Liver transplant			
Prevalence rate (%)	8 (50%)	8 (50%)	0.501
Fatality rate (%)	62.5%	12.5%	**0.006**
Kidney transplant			
Prevalence rate (%)	9 (39.13%)	14 (60.87%)	0.501
Fatality rate (%)	44.44%	0%	**0.039**
Solid organ transplant			
Prevalence rate (%)	17 (43.58%)	22 (56.42%)	0.501
Fatality rate (%)	52.94%	4.54%	**0.001**

**Note:** Number and percentage; mean and standard deviation; median and range.
